# Organ-Specific Hypereosinophilic Syndrome Presenting as Eosinophilic Gastroenteritis

**DOI:** 10.7759/cureus.16021

**Published:** 2021-06-29

**Authors:** Aneesh Kumar, Asim Haider, Ayesha Siddiqa

**Affiliations:** 1 Internal Medicine, BronxCare Hospital Center, Bronx, USA; 2 Internal Medicine, BronxCare Health System, Bronx, USA

**Keywords:** hypereosinophilia syndrome, eosinophilic gastroenteritis, eosinophilia, diagnosis of exclusion, eosinophilic gastrointestinal disorder

## Abstract

Gastrointestinal (GI) diseases have a substantial impact on the population health and healthcare resources of the United States. They constitute billions of dollars in expenditure and millions of office and hospital visits. With advancing diagnostic and treatment modalities, rare diseases are increasingly recognized and managed. However, after close to 80 years since the first description, eosinophilic GI disorders (EGID) are still uncommon, and only around 300 cases have been reported to date. Hypereosinophilic syndrome (HES) is well studied, but there are still no guidelines to direct management. We report the case of a 56-year-old female who presented with gastroenteritis and a persistent eosinophil count above 7 x 10^9^/L. Imaging was suggestive of bowel wall thickening, and endoscopy revealed normal-appearing mucosa. However, histologic examination revealed eosinophilic infiltration of the GI tract. She was diagnosed with HES and treated with oral prednisone with remarkable improvement of her symptoms.

## Introduction

Hypereosinophilic syndrome (HES) is a rare and under-reported group of diseases that are characterized by persistent eosinophilia above 1.5 x 10^9^/L on at least two occasions and eosinophil-mediated organ damage/dysfunction. Other causes of organ damage and secondary causes of hypereosinophilia must be ruled out [1]. Recognized variants of HES include myeloproliferative, T lymphocytic, familial, organ-restricted, specific/defined syndrome-associated, and idiopathic HES [2]. Eosinophilic gastrointestinal disorders are a group of inflammatory conditions characterized by eosinophilic infiltration of the bowel wall [3,4]. EGID can occur as an isolated disorder associated with hypereosinophilic syndrome (HES/EGID overlap) or can be part of a multisystem HES. The clinical presentation and management of both groups are remarkably similar [5].

## Case presentation

A 56-year-old female presented to the emergency room with abdominal pain, nausea, vomiting, and diarrhea. She reported intermittent, diffuse, non-radiating, crampy pain, which started around two weeks before. On a standardized pain scale, she rated it as high as 7 out of 10. She reported associated episodes of watery diarrhea five to six times a day and non-bloody non-bilious vomiting two to three times daily. She denied a history of fever, chills, recent travel, sick contact, weight loss, joint pain, cough, sore throat, runny nose, itching, and skin changes. She had presented two weeks prior with similar symptoms and was prescribed a seven-day course of oral metronidazole and ciprofloxacin, with a slight improvement of her symptoms. Her past medical history was significant for ductal carcinoma in situ of her right breast. Her surgical history included right-side mastectomy and cholecystectomy. She was allergic to penicillin, ibuprofen, and seafood, with unclear reactions. No significant family history was reported; she lives at home with her family and works as a school bus driver assistant. No toxic habits (e.g., drug abuse or smoking) were reported.

At the presentation, her vitals were stable. Examination revealed a middle-aged female in mild distress. Her abdomen was soft and non-distended, with mild diffuse tenderness. There was no evidence of free fluid or organomegaly. Rectal examination showed an empty rectal vault. No skin lesions or lymphadenopathy were noted. Heart and breath sounds were normal. Laboratory tests were significant for a leukocytosis of 33,300 cells/μl, with 75% eosinophils and an absolute eosinophil count of 24,900 cells/μl. Work-up including hemoglobin, platelet count, lipase, amylase, tryptase, ANA, IgE, and IgG were within the normal range (Table 1). The blood smear was negative for any blast cells. JAK2 mutation, flow cytometry, and human immunodeficiency virus (HIV) combination assay were negative. Stool analysis for ova and parasites was negative on three occasions. Serology for strongyloides IgG was negative.

**Table 1:** Patient's general hematology laboratory values WBC, White blood cells.

	Patient’s values	Reference range
Hemoglobin	12.4	12.0-16.0 g/dL
Platelet	325	150-400 k/µL
WBC	33.9	4.8-10.8 k/µL
Neutrophils	9.6%	40.0%-70.0%
Lymphocytes	9.2%	20.0%-50.0%
Monocyte	2%	1.0%-8.0%
Eosinophils	78%	≤5.0%

**Figure 1:**
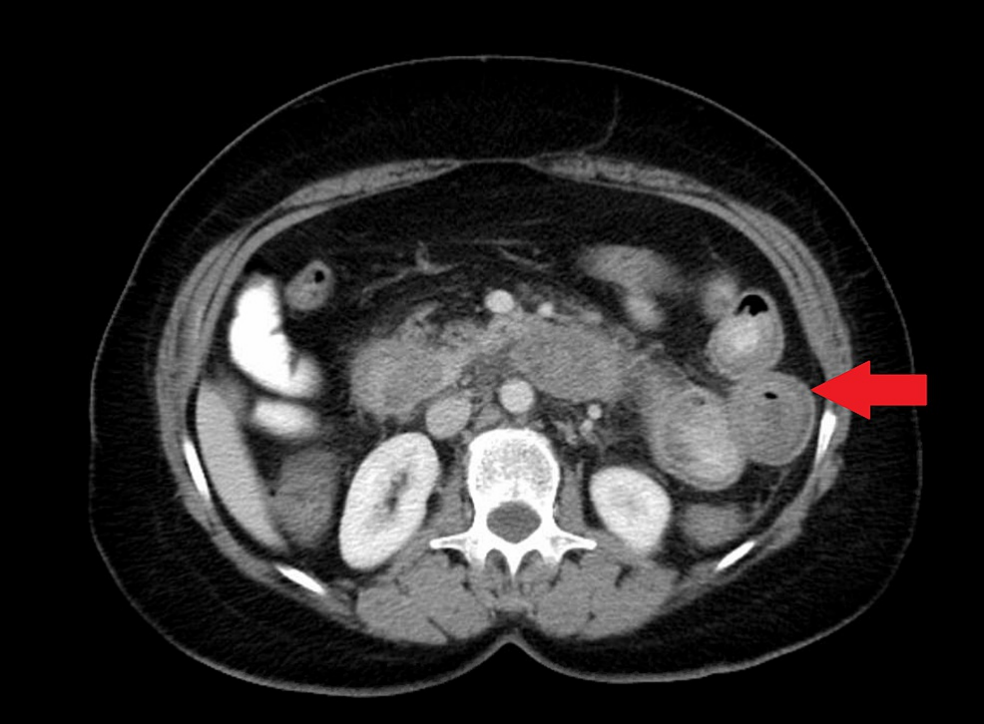
Computerized tomography abdomen and pelvis showing segmental wall thickening of the ileum suggesting enteritis

Computerized tomography (CT) of chest, abdomen, and pelvis with oral and intravenous contrast showed mild circumferential wall thickening within the distal esophagus and segmental wall thickening of the duodenum, proximal jejunum, and distal ileum, suggesting enteritis (Figure 1).

**Figure 2:**
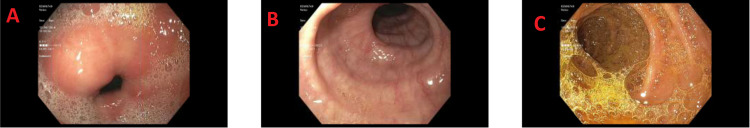
Esophagogastroduodenoscopy showing normal pylorus (A), esophagus (B), and duodenum (C)

The gastroenterology service evaluated the patient and performed endoscopy and colonoscopy, which was negative for any lesions, mass, polyps, or inflammation (Figure 2). Histopathology examination of the stomach, duodenum, and recto-sigmoid colon biopsy revealed increased eosinophilic infiltrate of lamina propria. Bone marrow biopsy showed hypercellular bone marrow (60%) with increased eosinophils, 52.6% of the total, and no evidence of abnormal myeloid maturation, increased blast population, or lymphoproliferative disease.

The patient was started on oral prednisone at 60 mg/day and significantly improved follow-up in three weeks. Repeat eosinophil count after three weeks was 7,300 cells/µl. Prednisone was tapered off and discontinued over the next few weeks. The patient is currently being monitored periodically for symptoms. 

## Discussion

Hypereosinophilia is defined as peripheral absolute eosinophilic count > 1.5 x 10^9^/L with or without end-organ damage. Common etiologies leading to hypereosinophilia include a wide range of allergic, infectious (parasitic and fungal), inflammatory, and neoplastic disorders. Our patient presented with elevated peripheral eosinophil count along with bone marrow and GI infiltration. However, extensive work-up to determine the underline etiology of hypereosinophilia in our patient was negative.

HES is truly a heterogeneous condition. The actual incidence and prevalence of HES are unknown. Based on a surveillance study, the highest estimated age-adjusted incidence rate was 0.18 per 100,000 person-years, and prevalence was 6.3 per 100,000 person-years, with a predominant male affliction (risk ratio: 1.47) [6]. The usual age of presentation is between 20 and 50 years, but cases have been reported in children. The clinical presentation of HES is highly variable, ranging from no symptoms to life-threatening cardiovascular or neurological complications, depending on the organ system involved. In around 6% of the patients, the onset of symptoms is insidious, and eosinophilia is detected incidentally. The presenting symptoms, in order of relative frequency, are dermatologic (37%), pulmonary (25%), gastrointestinal (14%), rheumatologic (7%), cardiac (5%), constitutional (5%), incidental (5%), neurologic (4%), and hematologic (3%) [7].

Since as early as 1937, eosinophilic involvement of the GI tract has been recognized. The prevalence of EGID is estimated to be around 18 per 100,000 persons. The pathogenesis of EGID includes a complex interaction between host cells, cytokines interleukin 3 and 5, granulocyte-macrophage colony-stimulating factor, the eotaxin chemokines, and mast cells. The clinical features of EGID differ based on (a) the layers of bowel involved, (b) location in the GI tract, and (c) degree of infiltration [6-8]. Based on the Klein classification, mucosal type EGID can present with dyspepsia, abdominal pain, nausea, vomiting, diarrhea, protein-losing enteropathy, or malabsorption. Muscular involvement can present with gastric outlet obstruction, pseudoachalasia, intestinal obstruction, strictures, or intussusception. Serosal involvement can present with ascites, peritonitis, or perforation [9-11].

There are no societal guidelines to aid the diagnosis of EGIDs. Evaluation includes a detailed history, examination, laboratory tests, histopathologic examination, and exclusion of secondary causes of eosinophilia [10]. History should investigate drug and food allergies, family history of allergies and other atopic diseases, travel history, drug history, and risk factors for malignancy. Laboratory tests are usually focused on the evaluation of eosinophilia [11]. Radiography is nonsensitive, nonspecific, and variable. Due to small bowel wall thickening that can be detected by ultrasound, some cases have been diagnosed by percutaneous biopsy. Endoscopy is also nonspecific and variable. It can range from normal-looking to ulcerated, friable, polypoid, strictures and even perforation. The cornerstone of diagnosis is histology. Multiple biopsies should be taken due to patchy involvement of the bowel. Specimens should be obtained from affected sites and healthy-looking sites to increase yield [12-14].

The treatment of HES is tailored to the underlying organ involved. A trial of the six food elimination has been studied. One meta-analysis reported significant symptomatic improvement but no histologic changes. Prednisone is the steroid of choice. It can induce remission of symptoms in two to three weeks and is rapidly tapered off in two weeks, although different tapering regimens have been studied. Relapses are common and may require repeat dosing or a long-term maintenance regimen. Table 2 quickly summarizes the other available options [15-19]. Our patient was diagnosed with HES and EGID and quickly responded to steroids. She gets routinely monitored for recurrence of GI symptoms and secondary organ involvement due to HES. 

**Table 2:** Summary of suggested medications for HES, their target, and mechanism of action PPIs, Proton pump inhibitors; LT, leukotriene; IL, interleukin; HES, hypereosinophilic syndrome. Source: References [15-19].

Medication	Target of action	Mechanism of action
Budesonide	Steroid receptors	Reduces inflammation
Montelukast	LT receptor	Reduces LT-mediated chemotaxis and recruitment
Cromolyn	Mast cell	Reduces the release of histamine and LTs
PPIs	IL-4 and IL-15	Reduces eosinophilia by receptor blockade
Azathioprine	DNA structure	Immunosuppression by cytotoxicity
Benralizumab	IL-5 receptor	Eosinophil recruitment and survival
Lirentelimab	Siglec	Induction of eosinophil cell death. Reduced mast cell activation

## Conclusions

Absolute eosinophil counts above 1.5 x 10^9^/L should warrant investigation for underlying etiology and surveillance for secondary organ involvement. Due to a myriad of presentations, eosinophilic gastroenteritis should be included in the differential diagnosis of unexplained gastrointestinal symptoms, especially in the light of peripheral eosinophilia. Short courses of steroids are the cornerstone of management. Novel steroid-sparing therapy should be attempted if one encounters steroid failure or relapse.
